# A Field-Applicable Method for Bluetongue Virus Detection Using Filter Paper Cards

**DOI:** 10.3390/vetsci13090955

**Published:** 2026-09-13

**Authors:** Estefanía Quiroga, Julieta Suyay Roldan, Nancy Cardoso, Juan Manuel Sala, Stefanía Selene Marucho, Cecilia Ferrufino, María José Dus Santos

**Affiliations:** 1 Instituto de Biotecnología, Universidad Nacional de Hurlingham, Villa Tesei 1686, Provincia de Buenos Aires, Argentina; quirogaestefania8@gmail.com (E.Q.); ferrufino.cecilia@inta.gob.ar (C.F.); 2 Instituto Nacional de Tecnología Agropecuaria (INTA), Instituto de Virología e Innovaciones Tecnológicas (IVIT), Nicolas Repetto y De los reseros s/n, Hurlingham 1686, Buenos Aires, Argentina; roldan.julieta@inta.gob.ar (J.S.R.); cardoso.nancyp@gmail.com (N.C.); marucho.stefania@inta.gob.ar (S.S.M.); 3 Consejo Nacional de Investigaciones Científicas y Técnicas (CONICET), Ciudad Autónoma de Buenos Aires 1425, Argentina; 4 Estación Experimental Agropecuaria Mercedes, Instituto Nacional de Tecnología Agropecuaria (INTA), Mercedes 3470, Provincia de Corrientes, Argentina; sala.juan@inta.gob.ar

**Keywords:** Bluetongue virus, filter paper cards, diagnosis, biosecurity, sample conservation

## Abstract

The diagnosis of Bluetongue Virus (BTV) in Argentina is primarily conducted via RT-qPCR and faces challenges due to the limited number of diagnostic laboratories, many of which are located in regions where BTV is exotic and far from primary sample collection areas. As a result, blood samples must be transported over considerable distances for analysis. This poses significant logistical challenges due to the specific transport conditions that are required. Consequently, the development of methodologies that optimize sample collection, preservation, and safe transport is of vital importance. The objective of this study was to standardize a sampling protocol for the detection of BTV using filter paper cards. This protocol was developed to ensure adequate preservation and transport of blood samples for diagnosis. Hydration with TE at 37 °C resulted in the lowest Cq among the tested treatments, with ~10 TCID_50_/mL representing the lowest viral concentration that was consistently detected under the experimental conditions. The stability of the sample on the cards was evaluated through a series of storage trials at temperatures of 25 °C, 4 °C, −20 °C, and −80 °C for 1, 7, 30, 60, 120, and 360 days. Stability was verified up to 120 days at all temperatures. Furthermore, the absence of infectious material in the card eluate was confirmed. A preliminary field evaluation was conducted, in which blood samples from cattle and sheep were collected in tubes and on cards. According to the Cohen’s Kappa index, the agreement between both methods was nearly perfect. The standardized methodology signifies a substantial advancement in BTV diagnosis, as it facilitates the transportation of samples without the necessity of refrigeration and with adequate biosafety conditions without losing diagnostic capacity.

## Simple Summary

The diagnosis of Bluetongue Virus (BTV) in Argentina is predominant reliant on RT-qPCR, but it is challenged by the scarcity of diagnostic laboratories and the considerable distances between sampling sites and testing facilities. These logistical constraints underscore the necessity for simple and reliable methods for sample preservation and transport. The objective of this study was to standardize a filter paper-based sampling protocol for BTV molecular diagnosis. Hydration with TE at 37 °C provided optimal analytical performance, yielding ~10 TCID_50_/mL as the lowest viral concentration consistently detected under the experimental conditions. The sample stability was assessed after storage at temperatures of 25 °C, 4 °C, −20 °C, and −80 °C for up to 360 days. The results demonstrated that diagnostic stability was maintained up to 120 days under all conditions tested. The absence of infectious material in card eluates, substantiates their biosafety with regard to transport and handling. A preliminary field evaluation using blood samples from cattle and sheep showed nearly perfect agreement between card-based and conventional sampling (Cohen’s kappa). This standardized approach offers a practical and safe alternative for BTV surveillance, enabling sample transport without refrigeration while preserving diagnostic performance.

## 1. Introduction

Bluetongue (BT) is a viral disease that is primarily transmitted by hematophagous vectors of the genus *Culicoides*. According to the World Organization for Animal Health (WOAH) [1] and the National Agrifood Health and Quality Service of Argentina (SENASA) [2] BT has been classified as a notifiable disease. The sanitary and economic impact of BT involves loss of body condition, reduced milk yield, infertility and abortion, alongside indirect costs associated with international trade restrictions and the surveillance requirements imposed to limit disease spread [3,4].

Bluetongue virus (BTV), the etiologic agent of BT, is the prototype member of the *Orbivirus* genus (Sedoreoviridae family) [5]. BTV has been identified as a pathogen that infects both domestic and wild ruminants. However, the disease manifests with greater severity in sheep and white-tailed deer. Clinical signs have also been observed in cattle, goats and camelids [6,7,8]. Although often unapparent, infection in these other species allows them to act as reservoirs, remaining a prolonged period of viraemia for several months, particularly in cattle [9].

Worldwide, at least 30 BTV serotypes have been identified [8,10]. Of these, 27 are notifiable to the WOAH [4]. In Argentina, only two serological surveys were conducted in the mid-1990s. Subsequently, BTV isolates were reported in 2001, 2009, and 2010 [11,12,13] from bovines and sheep in the north of Corrientes and Misiones provinces. The isolates in question corresponded to the serotype 4 and exhibited a strong similarity to those previously identified in Brazil, thereby suggesting a common origin [12]. The extant evidence suggests that BTV circulates throughout South America, with only natural barriers potentially limiting further spread [13].

The diagnosis of BTV is made by laboratory analysis, with RT-qPCR being the most common technique due to its rapid and sensitive diagnostic capabilities [1]. Given its status as a notifiable agent, the transportation of biological samples from suspected cases is of paramount importance, requiring both adequate material preservation and strict biosafety compliance. Furthermore, transport must adhere to national, regional, and international regulations to minimize the risk of exposure and accidental virus dissemination, especially when samples are transported to BTV-free zones. Consequently, the development of methodologies that optimize blood sampling, conservation and transportation is of paramount importance.

A valuable alternative to address this issue is the use of filter paper cards (FPC), which are specifically designed to stabilize nucleic acids stable at different temperatures. This allows for transportation without refrigeration [14,15].

This technology employs filter paper that has been impregnated with a proprietary blend of chemical denaturants and a free radical scavenger. The mixture has been demonstrated to lysate cells and organelles, inhibit bacterial overgrowth, and denature proteins. Concurrently, the scavenger functions to protect nucleic acids from damage caused by free radicals. During the process, proteins and inhibitors are eliminated, while the RNA remains sequestered within the matrix [16].

The immediate inactivation of microorganisms present in the samples applied to filter paper renders the system entirely safe [17]. FPCs have found widespread application in numerous fields, including biobanking, forensic genetics, and molecular epidemiology research, due to their capacity to preserve nucleic acids over extended periods [14]. The efficacy of these methods in detecting RNA viruses has been well-documented. Examples of the viruses that have been detected include foot-and-mouth disease virus (FMDV), avian influenza virus (AIV), porcine reproductive and respiratory syndrome virus (PRRSV), bovine viral diarrhea virus (BVDV), rotavirus (RV), African swine fever virus (ASFV), West Nile virus (WNV), rubella virus, measles virus, and human immunodeficiency virus (HIV) [18,19,20,21,22,23,24,25,26,27,28,29,30,31]. Recent studies have also evaluated the use of FPCs for SARS-CoV-2 detection. These studies demonstrated that the cards are safe (regarding infectivity), preserve viral RNA, and allow for room temperature transport [32,33].

This technology is user-friendly and requires only a small sample volume applied to the filter paper, potentially allowing the use of less invasive blood collection methods under field conditions. Moreover, the implementation of this technology does not necessitate the use of specialized equipment or the expertise of trained personnel [34].

The objective of this study was to standardize a BTV sampling protocol using FPCs to ensure adequate preservation and transport of blood samples for subsequent diagnosis.

## 2. Materials and Methods

### 2.1. Viral Stock

A BTV stock (L01/23, strain 4/ARG/99/2001) with a titer of 1 × 10^6^ TCID_50_/mL was used. The viral stock was provided by the Institute of Virology and Technological Innovations (IVIT), INTA. The stock was independently quantified by RT-qPCR assay employed in this study, yielding 2.75 × 10^8^ genome copies/µL (Supplementary Material S1).

### 2.2. Filter Paper Cards

GenSaver™ 2.0 Cards (Ahlstrom-Munksjö, Ahlstrom Germany GmbH, Niederschlag, Germany) were used throughout the study.

### 2.3. Bovine Blood

Blood was obtained from a bovine at the Research Center for Veterinary and Agronomic Sciences (CICVyA) experimental field and tested negative for BTV by RT-qPCR. Blood collection was performed according to the procedure approved by the Institutional Committee for the Care and Use of Experimental Animals (CICUAE 29/2022).

### 2.4. RT-qPCR for BTV Genome Detection

The RT-qPCR assay was based on the method described by Maan et al. [35] and amplifies a 93 bp fragment of BTV segment 9. The primer and probe sequences are shown in Table 1.

For the reaction mixture, the iTaq Universal Probes One-Step kit (BIORAD) was used, containing each primer and the probe at a concentration of 1 µM and 0.25 µM, respectively. The final reaction volume was 10 µL, containing 7.5 µL of reaction mix and 2.5 µL of template. Reactions were run on ABI7500 (Thermo Fisher Scientific Inc., Waltham, MA, USA) or CFX96 (Bio-Rad Laboratories, Inc., Hercules, CA, USA) real time PCR equipment. The thermal profile used was:

50 °C, 10 min; 95 °C, 3 min followed by 40 cycles of 30 s at 95 °C and 1 min at 60 °C.

When the reaction template was viral RNA, it was previously denatured by heating a suitable volume of RNA in a 0.2 mL PCR tube at 95 °C for 5 min in a Simpliamp thermal cycler (Thermo Fisher Scientific Inc., Waltham, MA, USA). The tubes were then immediately placed on ice for at least 5 min. Denatured RNA was incorporated into the reaction mix. Each reaction included a non-template control and a positive control (RNA from viral stock diluted 1/1000). The cut-off used to classify a sample as detectable corresponded to a Cq ≤ 40.

A plasmid was generated containing the sequence of BTV segment 9 to be used as a standard in calibration curves for viral genome quantification. The segment 9 amplification was performed via end-point RT-PCR using viral RNA as the initial template. The 1045 bp amplification product was inserted into the pGEM T Easy vector (Promega Corporation, Madison, WI, USA), and its sequence was verified by Sanger sequencing.

The technique used has an LOD and LOQ of 3.15 genetic copies (gc) per reaction.

### 2.5. Standardization of the RNA Elution from Filter Cards

To evaluate different treatments, 120 µL of a sample containing 30 µL of virus and 90 µL of bovine blood was seeded on the cards in quadruplicate in a Type 2 biosafety cabinet (Telstar, Terrassa, Barcelona, Spain). They were allowed to dry at room temperature and then stored at −80 °C until processing. A hole puncher was used to obtain approximately three 6 mm punches for each sample. The puncher was decontaminated after processing each sample to prevent cross-contamination. Experimental conditions were established based on literature review and are summarized in Table 2. In all cases, the TIANamp Virus RNA Kit (TIANGEN Biotech (Beijing) Co., Ltd., Beijing, China) was used for viral RNA purification. Viral RNA (positive control) and RNA from uncontaminated blood (negative control) were included. The method that yielded the best RT-qPCR detection results was selected.

### 2.6. Analytical Sensitivity of BTV Detection from Filter Cards

Serial dilutions of the virus in bovine blood were prepared from 1 × 10^2^ to 1 × 10^5^ genetic copies/µL (gc/µL). At least three replicates of each dilution were seeded on filter cards in a II-BSC, allowing 2 h of drying in the cabinet followed by 24 h at −80 °C. Parallel processing was performed for contaminated blood dilutions with and without the card step, performing RNA extraction with prior hydration in TE buffer for 30 min at 37 °C. Samples were then run in RT-qPCR with a standard curve (2.5 × 10^7^ to 2.5 × 10^2^ gc/µL) to quantify genetic material from the blood and the cards.

### 2.7. Cell Inoculation Test: Control of the Inactivation of BTV Trapped on Filter Cards

To assess whether BTV infectivity remained detectable after card processing, viral stock, BTV-spiked blood samples and medium were applied to the cards (Table 3). For the card loading, 45 µL of the sample were placed onto the designated circles of the card in a II-BSC and allowed to dry for two hours. Cards were then stored for 24 h at −80 °C. Punches were extracted in the same cabinet used for seeding, ensuring sterility with a hole puncher. Since it has been reported that macerates from GenSaver 2.0 cards induce cytotoxicity in Vero cells [17], the macerates were diluted in PBS before use. Samples were placed in 2 mL tubes with 1 mL of PBS and homogenized by gentle vortexing. They were incubated at 4 °C for 2 h and centrifuged at 1500 rpm for 10 min Dilutions of the resulting eluate were prepared in DMEM supplemented with 2% fetal bovine serum (FBS) (Internegocios, Buenos Aires, Argentina). T25 flasks containing Vero cells at 80% confluence were infected using 500 µL of inoculum. The cells were incubated at 37 °C under a 5% CO_2_ atmosphere and agitated every 15 min for 1 h. Subsequently, 5 mL of DMEM with 2% FBS were added, and the cultures were incubated at 37 °C for 2 days until the next passage. Three successive passages were conducted in Vero cells, with cultures monitored microscopically for cytopathic effect (CPE). Finally, RT-qPCR was performed to verify the absence of viral RNA in cells inoculated with the card elutions. These viral amplification trials were conducted in a WOAH-certified BSL-4 laboratory.

### 2.8. Stability of Viral RNA on the Filter Cards

To evaluate the stability of BTV genetic material on the cards, four storage temperatures were evaluated over 360 days. Cards were seeded with spiked bovine blood in two conditions: Condition 1 represents a 1/10 dilution of the viral stock (1 × 10^5^ TCID_50_/mL) and Condition 2 represents a 1/1000 dilution of the same viral stock (1 × 10^3^ TCID_50_/mL). 45 µL per sample were seeded in three 15 µL spots, with 5 replicates per sample in a II-BSC and allowed to dry for two hours. The dry inoculated cards were placed in plastic bags with desiccant and kept at different temperatures (Table 4). At the end of each storage period, viral genome detection and quantification were performed.

### 2.9. Field Trial

Sampling was conducted at two different farms with suspected BTV circulation in the Virasoro department, Corrientes province. Blood was collected from the jugular vein using a syringe; one drop was seeded on cards, while the remaining volume was placed in tubes with anticoagulant. Samples were transported by land, simulating a routine diagnostic procedure. Each field sample was analyzed in triplicate by RT-qPCR for both direct blood and FPC samples. A Cq cut-off of 40 was used, and samples were classified as detectable only when all three replicates showed amplification with Cq ≤ 40. Blood and card samples were processed simultaneously, and results were compared.

### 2.10. Statistical Analysis

As dependent variables were continuous quantitative in nature, assumptions of homogeneity of variance and normality were evaluated. Subsequently, a General Linear Model was fitted, and orthogonal contrasts with Bonferroni correction were performed.

Since independent filter paper cards were processed at each sampling time in the stability study, observations were considered independent. The effects of storage time and temperature on viral genome stability were therefore evaluated using a two-way factorial general linear model, including time, temperature, and the time × temperature interaction. Analyses were performed separately for each viral concentration.

The agreement between the two viral detection techniques was assessed using Cohen’s Kappa index. This statistic was chosen as both methods were treated as independent assays without a predefined gold standard. All analyses used InfoStat software, version 2020.

## 3. Results

### 3.1. Comparison of Filter Paper Card Processing Conditions for Subsequent BTV RT-qPCR Detection

Initially, a literature search was conducted to select the conditions for experimental evaluation [21,35,36]. In light of the aforementioned findings, a total of six distinct treatments were subjected to evaluation with the objective of extracting nucleic acids from the cards. Specifically, these conditions accounted for incubation temperature, post-hydration time, hydration buffer type, and agitation.

The mean Cq values obtained from Condition 1 (hydration with TE at 37 °C for 30 min) were the lowest mean Cq value among the conditions evaluated (GenSaver 2.0; bovine blood). Significant differences were observed compared with conditions No. 3, 4, and 6 (*p* = 0.0113; *p* = 0.0311; *p* = 0.0008, respectively) (Table 5; Supplementary Material S2). Although Condition 5 (Chelex) yielded comparable results in RT-qPCR, Condition 1 was selected for subsequent experiments because of its lower mean Cq, under the experimental conditions that were tested, and its greater methodological simplicity. Because absolute RNA recovery was not determined, differences in Cq values cannot be attributed exclusively to differences in RNA release/recovery from the cards, as differential effects of the tested conditions on RT-qPCR inhibition cannot be excluded. Therefore, the comparison should be interpreted in terms of the overall RT-qPCR performance of the different card-processing conditions rather than absolute RNA recovery efficiency.

### 3.2. Analytical Sensitivity of the FPC/RT-qPCR Methodology

**Figure 1:**
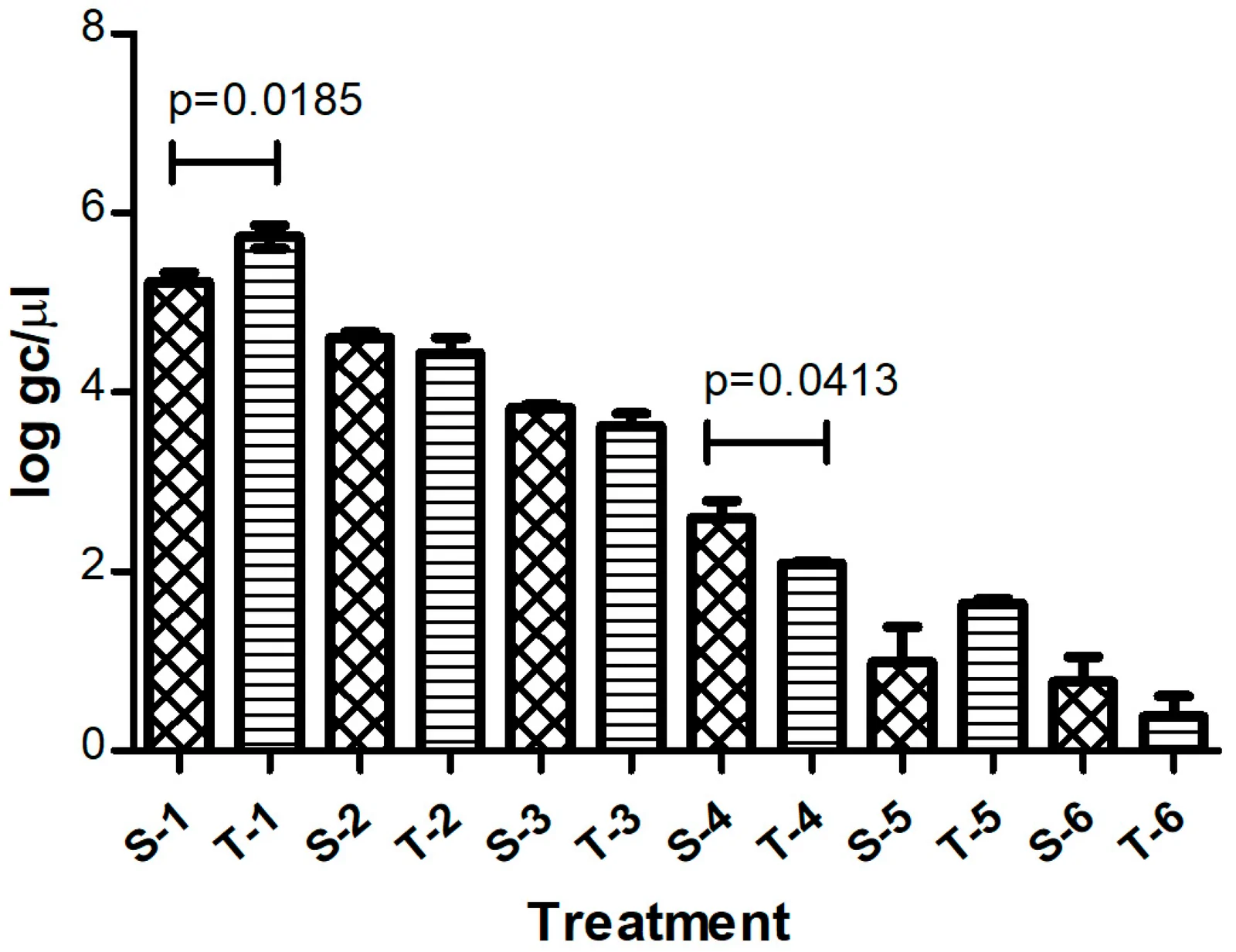
BTV detection by RT-qPCR from virus-spiked bovine blood samples (S, cross-hatched bars) and virus-spiked bovine blood samples applied to filter paper cards (T, horizontally striped bars). Starting from a viral stock (2.75 10^8^ gc/µL), serial 10-fold dilutions were prepared in bovine blood: 1/10 (−1), 1/100 (−2), 1/1000 (−3), 1/10,000 (−4), 1/100,000 (−5), and 1/1,000,000 (−6). The spiked blood was processed directly, while the blood previously applied to the cards was analyzed using the standardized procedure developed in this work. In each case, viral genome copies per µl of reaction (gc/µL) were quantified.

Six serial dilutions (ranging from 10-1 to 10-6 gc/µL) of BTV in a blood matrix from healthy donors were utilized as surrogates for clinical samples. The mean viral loads (VLs) obtained from FPC and blood specimens were then compared. Significant differences were observed between the genetic copies detected in blood and those detected on cards exclusively at the 1:10 dilution (*p* = 0.0185) and a marginal difference at the 1:10,000 dilution (*p* = 0.0413). Furthermore, the viral genome was detected in both treatments up to the 1:100,000 dilution, corresponding to an approximate titer of 10 TCID_50_/mL based on the initial titer of the viral stock and the dilution factor (Figure 1, Supplementary Material S3). In the context of the experimental conditions, both the card-based and direct blood RT-qPCR approaches exhibited a consistent ability to detect the viral genome at the lowest tested concentration.

### 3.3. Assessment of BTV Inactivation on the Filter Paper Cards

In order to assess the safety of the cards regarding BTV inactivation, a viral infectivity assay was conducted. The presence of CPE was observed exclusively in the positive control (V1) and the spiked blood sample that had not undergone processing through the card (M1). These findings provide evidence in support of a loss of detectable BTV infectivity under the experimental conditions tested. In accordance with these findings, the viral genome was detected by RT-qPCR exclusively in the V1 and M1 samples. CN1 and CN2 samples served as controls for cell viability and to assess any potential toxicity arising from the card processing (Table 6).

### 3.4. Long-Term Storage Evaluation of Nucleic Acid in FPC

To evaluate the preservation of genetic material on the cards, a stability assay was performed using bovine blood samples spiked with two distinct viral titers (1:10 and 1:1000 dilutions of the viral stock, corresponding to 10^5^ and 10^3^ TCID_50_/mL, respectively).

**Figure 2:**
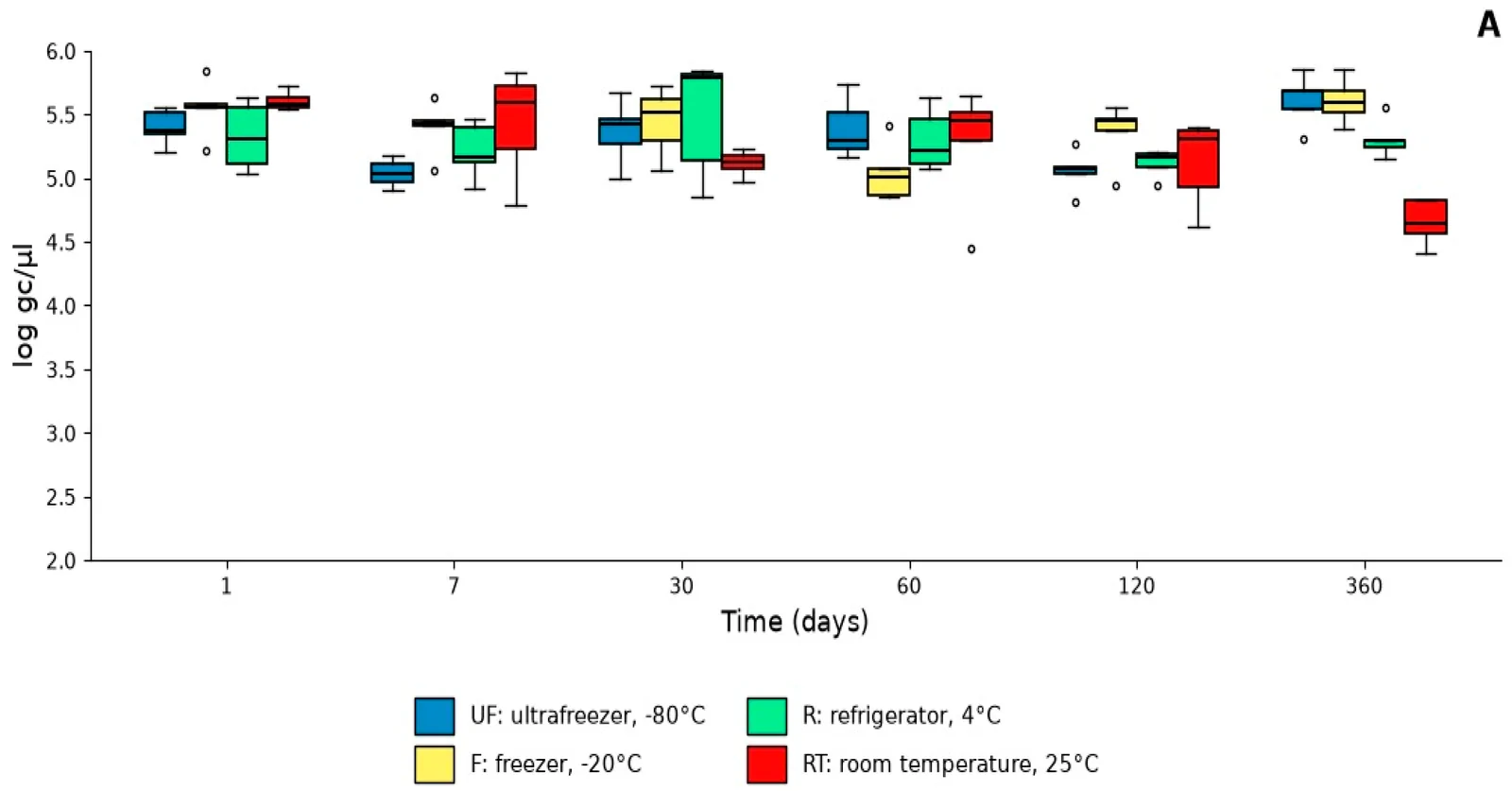

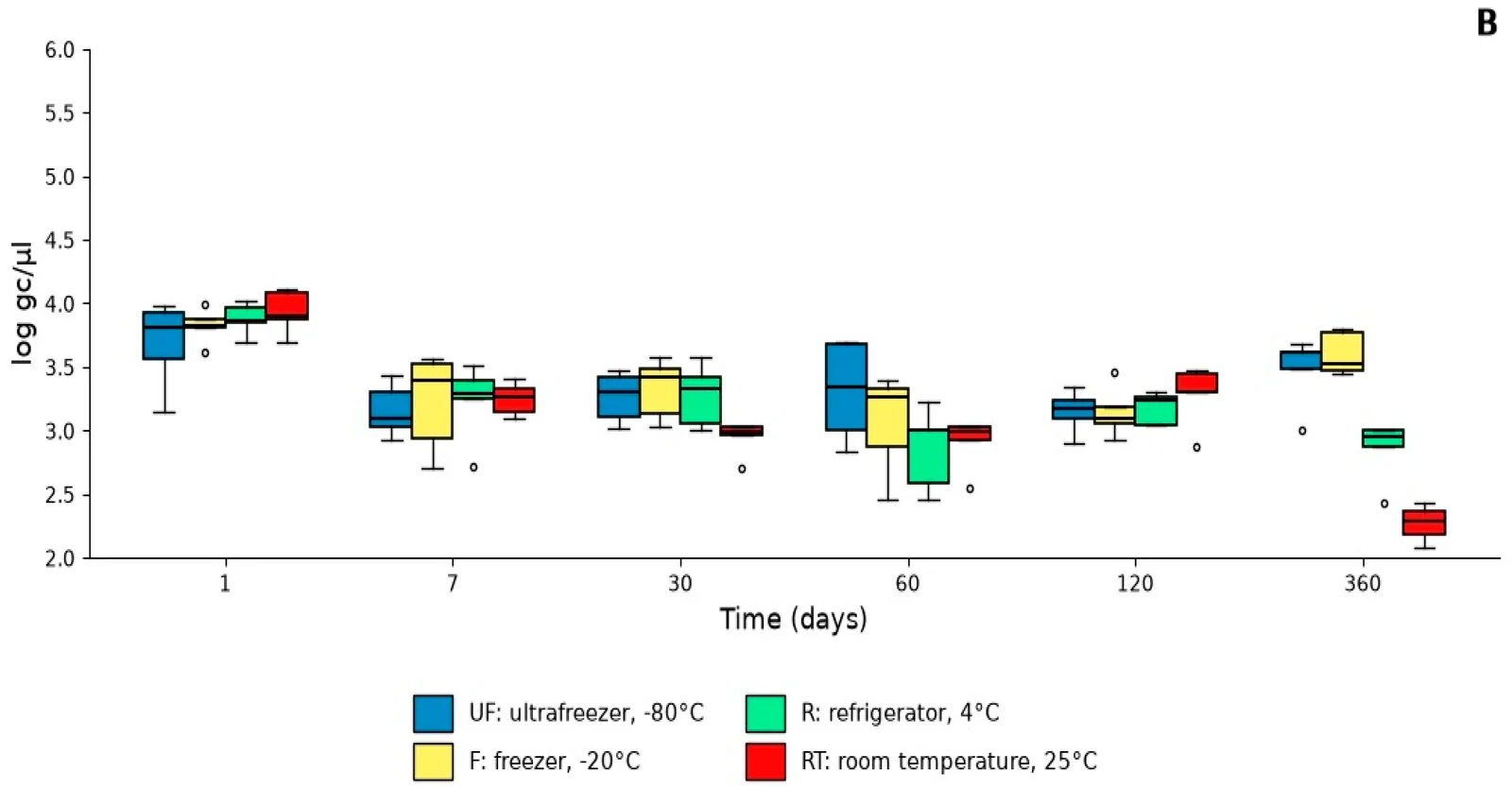
Stability of BTV RNA on filter paper cards stored at different temperatures for 360 days. Box-and-whisker plots show the genome copies/µL over time for the 1:10 dilution (10^5^ TCID_50_/mL) (**A**) and the 1:1000 dilution (10^3^ TCID_50_/mL) (**B**). Colors indicate the different temperatures analyzed. UF: Ultra-freezer (−80 °C); F: Freezer (−20 °C); R: Refrigerator (4 °C); RT: Room temperature (25 °C).

In the evaluation of the most concentrated samples (Figure 2A), genome copies remained at similar levels over time, with only minor variations between time points. At 360 days, the variation between treatments increased, particularly for samples stored at room temperature (25 °C), which showed significant differences compared to the other conditions. Nevertheless, genome copy values for samples stored at 4 °C, −20 °C, and −80 °C remained stable (Figure 2, Table 7, Supplementary Material S4).

Regarding the samples of a more diluted nature, there was a slight decrease in genome copies to 7 days post-loading, after which they remained stable until day 120. As illustrated in Figure 2A, a higher degree of variability was noted at 360 days for samples stored at room temperature in comparison to those maintained at the other temperatures (Figure 2, Table 7, Supplementary Material S4).

Furthermore, the factorial analysis revealed a significant interaction between storage time and temperature for both viral concentrations (*p* < 0.001). This finding indicates that temporal changes in viral genome recovery are contingent on the storage temperature (Supplementary Material S4).

These results confirm the stability of BTV RNA across all time points when stored at −80 °C, −20 °C, and 4 °C, and for up to 120 days when stored at room temperature, under low-humidity laboratory conditions.

### 3.5. Agreement Between FPC and Blood Specimens in BTV RNA Quantitation

A total of 48 blood samples from cattle and sheep were evaluated, originating from a sampling mission in Virasoro, Corrientes. Samples were collected in both anticoagulant tubes and filter paper cards. These samples were subsequently transported by land to the Laboratory, where they underwent a simultaneous processing procedure. The standard diagnostic protocol was followed for the blood samples, which involved RNA extraction followed by RT-qPCR; while the samples applied to the cards were processed using the methodology standardized in this study.

In this pilot dataset, which includes 48 paired samples from two farms, a high degree of agreement between the results of the filter paper card and the paired whole-blood RT-qPCR tests was observed (agreement: 93.75%; Cohen’s κ = 0.874; 95% CI, 0.735–1.000). This finding indicates a strong concordance under the conditions evaluated (Table 8, Supplementary Material S5). Three discordant results were obtained from the field samples (BOV LH29, LH43, and LH44). LH43 and LH44 were classified as non-detectable by direct blood RT-qPCR but detectable after FPC processing, whereas LH29 was detectable in direct blood but non-detectable after FPC processing (Supplementary Material S5). However, the limited sample size and restricted epidemiological setting preclude a comprehensive assessment of diagnostic performance. Therefore, these results should be interpreted as a preliminary field assessment of agreement with the paired whole-blood RT-qPCR method rather than as a full clinical validation. In order to establish the clinical diagnostic performance, it is necessary to conduct larger prospective studies that include a variety of epidemiological settings and appropriate stratification.

## 4. Discussion

In Argentina, the BTV is classified as a viral agent that is subjected to mandatory notification to sanitary authorities. Consequently, it is imperative to preserve samples until a diagnosis is made and to prioritize the acquisition of reliable results. As in the case with many arboviruses, BTV is predominantly present in tropical and subtropical regions where researchers and veterinarians often encounter challenges in maintaining and transporting biological samples within a continuous cold chain from the field to the analytical laboratory. In Argentina, the detection of this pathogen is predominantly conducted in areas where the virus is not present, requiring strict biosafety measures during sample collection and transport. Hence, FPCs emerge as a promising alternative facilitating the containment of numerous samples within a compact space and offering a straightforward mechanism for in situ sampling. In addition to inactivating pathogens and protecting the genetic material within the matrix, they minimize biological risk of exposure and reduce storage and transport costs for large-scale sampling due to their preservation at room temperature.

The utility of the recoverable RNA depends on the sample material, the type of nucleic acid (single- or double-stranded), the duration of storage, and the sensitivity of the assay employed [17]. In numerous studies, a variety of FPCs have been utilized for the collection and transportation of clinical and field samples for subsequent molecular detection, genotyping, and sequencing of the genes of several viruses, and even for recovering whole genomes. These include both enveloped and non-enveloped RNA and DNA viruses [25,26,30,37,38,39,40,41]. To the best of our knowledge, the present study is the first to develop a comprehensive BTV-specific FPC protocol. This protocol encompasses sample application, elution, and storage conditions, as well as a preliminary field evaluation. It is also noteworthy that, with the exception of one previous study comparing different FPCs for the preservation of various virus types, including BTV [17], no other studies have developed a complete BTV-specific FPC protocol.

When a sample is placed on the card, the matrix absorbs the genetic material. Therefore, finding an effective and simple RNA release method is of the outmost importance. In a previous study, our group demonstrated that BVDV RNA can be efficiently extracted from FPCs using TE buffer or DMEM medium at 37 °C [21]. Therefore, we compared this condition with various treatments selected from the literature [14,35,42]. During the optimization phase, recovery and assay inhibition were not quantified independently and lower Cq values were used as a surrogate for overall performance assessment. While analogous results were obtained across all evaluated conditions, the TE treatment at 37 °C (C1) yielded significantly different results. Consequently, it was selected for further analysis.

The evaluation of analytical sensitivity was conducted by employing serial dilutions of BTV in bovine blood, thereby simulating variations in viral load. The lowest viral concentration that was consistently detected, based on the initial viral stock titer and dilution factor, was approximately 10 TCID_50_/mL. This finding supports the feasibility of this methodology for testing samples from suspected BTV cases.

Previous studies using filter cards have reported a broad spectrum analytical sensitivity depending on the virus, card type, and detection method. The reported values for these viruses range from 0.0016 TCID_50_ for rabies virus [31] to 10^5^ TCID_50_/mL for FMDV [30], while sensitivities of 10^3^–10^5^ EID_50_/mL have been reported for AIV and NCV [19,27]. A direct comparison between these values and those obtained for BTV is precluded by the existence of significant discrepancies. These discrepancies are attributable to differences in viruses, experimental systems, detection methods, and filter card types.

Under our experimental conditions, BTV RNA was detected at the same lowest tested concentration in card-processed and direct blood samples. Similar findings have been reported for FMDV [30] and rabies virus [31]. On the other hand, reduced RNA recovery or analytical sensitivity after card processing has been described for NCV [27], CSFV, SV-A, ASFV, and SuNV1 [43]. For BTV specifically, Keck et al. (2022) [17] reported that GenSaver 2.0 and FTA^®^ cards provided optimal viral RNA recovery among the cards evaluated. GenSaver 2.0 cards have also shown good performance for Influenza A virus detection [35].

In animals infected with BTV, such as cattle, sheep, and goats, the peak of viremia occurs usually 7–10 days after infection and is sustained for an average of 50 days [44]. In experimental infection trials, Flannery et al. (2019) [45] reported that BTV-8 infection kinetics showed VL values in the range of 10^3^–10^9^ gc/mL at the viremia peak (5–9 days post-infection, dpi) and 10^3^–10^6^ gc/mL at 20 dpi. In the absence of an evaluation of viral load (VL), other experimental BTV infection studies have presented Cq values, which ranged from 18 to 27 at 7 dpi [46,47]. Recently, Newbrook et al. (2024) [48] observed that sheep infected with a serotype 3 isolate from Great Britain, exhibited an infection peak between 4 and 10 dpi, with an average VL of 9.24 log_10_ gc/mL of blood. At 28 dpi, VLs remained elevated, with values ranging from 5.99 to 7.66 log_10_ gc/mL of blood. In light of the aforementioned findings, we conclude that our diagnostic methodology can effectively detect the viral genome in viremic animals. We believe that the relevance of these results is attributable to the fact that blood constitutes a matrix capable of affecting PCR, and the seeding volume is typically minimal, resulting in a certain degree of material loss during the filtration process.

Currently, the transportation of blood samples is required to be conducted in refrigerated conditions at a temperature of 4 °C. Each sample must be placed in an anticoagulant-filled tube. On a large scale, this procedure requires the utilization of multiple refrigerated boxes that must be transported over extensive distances (often hundreds of kilometers) to specialized diagnostic laboratories situated in regions where the virus is considered exotic. Therefore, we argue that an adequate diagnostic methodology must encompass both material conservation and the biosafety requirements associated with sample handling. Several studies have demonstrated that various pathogens (including viruses and bacteria) are inactivated when passed through filter paper cards [14,16,27,30,31,36]. However, in a certain cases, some viruses have demonstrated resilience, maintaining their viability and infectivity after passing through specific cards [17,29,49].

To assess the infectivity of BTV after its passage through the card, a virus elution protocol was employed that had previously been described for arboviruses using the same cellular system [36]. As expected, BTV did not demonstrate infectiousness following card processing under the evaluated drying and storage conditions, irrespective of whether the virus was in culture medium or the blood matrix. Moreover, no cellular toxicity was observed during the course of this study. This finding is consistent with that reported by Keck et al. (2022) [17] when evaluating BTV inactivation on GenSaver2.0 cards and Vero cells.

In works aimed at optimizing the use of FPCs, Sakai et al. (2015) showed that the optimal storage temperatures for preserving RNA samples in the long term are 4 °C, −20 °C, and −80 °C [50]. In this study, we verified that viral RNA remains stable for up to 120 days at storage temperatures of −80 °C, −20 °C, 4 °C, and room temperature, and for up to 360 days if room temperature storage is omitted. According to Cardona-Ospina et al. (2019) [14], the storage of cards at 4 °C allowed the preservation of fragments of up to 1200 bp. Importantly, the fragment amplified by the RT-PCR in this study is less than 100 bp, a size that offers more favorable conditions for amplification. Other researchers found that temperatures of −20 °C and −80 °C exhibited superior preservation of the genetic material for Rabies Virus. Conversely, a substantial decrease in extraction rates was observed at 4 °C and room temperature [50]. In contrast, Kanta et al. (2022) [32] found that SARS-CoV-2 was detectable at 4 °C and 37 °C for up to 14 days. Furthermore, Krambrich et al. (2022) [36] reported that when evaluating the stability of alpha and flaviviruses on FTA^®^ cards, the RNA degraded in a temperature- and time-dependent manner, with 4 °C being the most stable condition. In the case of NCV-containing allantoic fluid samples on FTA^®^ cards, Perozo et al. (2006) [27] observed a 1 log_10_ decrease in sensitivity after 14 days of storage at room temperature.

It is important to note that, in our case, the preservation of genetic material at room temperature eliminated the necessity for low-temperature transportation, thereby reducing expenditures associated with refrigeration. However, it is important to evaluate all temperatures for periods exceeding 360 days to ascertain the percentage of RNA loss over prolonged periods. In addition, available sampling intervals do not allow a precise determination of when the decline between 120 and 360 days occurred. Since suspected bluetongue samples may originate from subtropical climates, further studies should consider the conservation at higher temperatures. Krambrich et al. (2022) [36] reported that viral RNA degraded rapidly when researchers fixed Sindbis virus and tick-borne encephalitis virus on FTA^®^ cards and stored them at 37 °C.

The feasibility of the detection method was evaluated through a field trial that utilized positive and negative samples collected from cattle and sheep. The detection process was replicated using parallel blood samples resulting in the identification of 27 out of 48 detectable samples on cards and 26 out of 48 in direct blood. Conversely, viral RNA was not detected in 22 out of 48 blood samples and 21 out of 48 FPC samples. These results suggest that our detection method yields performance comparable to direct blood processing. Notably, the viral loads (VLs) in the analyzed samples were low, with some approaching the limit of detection. Consequently, we acknowledge the necessity to increase the sample size and incorporate animals with varying VLs to clinically validate this methodology. Muthukrishnan et al. (2008) [30] also conducted a similar evaluation, analyzing vesicular fluid from the tongue epithelium of animals that were naturally or experimentally infected with FMDV. The authors demonstrated that conventional sampling and FTA^®^ cards produced identical results in virus detection and serotyping. In another study, Yacouba et al. (2020) [41] compared FTA^®^ cards for the analysis of HIV-1 VL with plasma testing and found no significant differences in the VLs determined by both methodologies. A limitation of the present study is that ambient conditions during field transport were not monitored. Furthermore, the performance after prolonged exposure to 35 °C and high humidity was not evaluated. Therefore, it is advisable to exercise caution when deploying in hot, humid climates.

When collecting samples, it is important to consider the volume of the sample to be deposited (in order to prevent the sample from breaking through the card) and the drying time. We have noted that sufficient space must be available for drying the cards, which is an important consideration when sampling in situ. It is important to acknowledge that operational factors such as early post-spotting intervals, high humidity, oversaturation volumes, packaging configurations, and real-world transport stresses (temperature/humidity fluctuations) were not evaluated and should be addressed in future research. The advantages of employing this methodology are manifold. Firstly, the refrigerated transport of samples is to be avoided, although the stability of the deposited material must be ascertained. Secondly, the required sample volume is minimal, thus enabling the expeditious sampling of animals and the transport of smaller bulk packages. This is of particular importance in locations with limited infrastructure and resources, as well as for large-scale surveys. Thirdly, the biological risk associated with the transportation of infectious samples is minimized. However, in the case of DNA genome viruses, the degree of inactivation resulting from contact with the cards may be partial. However, if the sample is correctly seeded, the risk of transmission is minimized because the card fibers immobilize the sample prior to initial drying. The flip-down protective cover then provides an additional layer of protection, followed by transport in a sealed bag. The addition of heat treatment, 0.2% acetic acid, or phenol is a viable option without affecting subsequent PCR analysis [17].

## 5. Conclusions

Our results indicate that, when combined with TE buffer elution, RNA purification, and RT-qPCR detection, FPCs can safeguard BTV genetic material thereby enabling subsequent molecular detection. We also confirmed that the BTV loses its infectivity in Vero cells upon passing through the filter paper matrix. Finally, through a pilot field sampling, we observed high agreement between cards and blood, supporting the applicability of the developed and standardized diagnostic methodology. Comprehensive validation under high heat/humidity and varied field logistics is required to confirm the results obtained.

This approach facilitates effective and simple sampling, thereby reducing the costs of large-scale operations by enabling safe transport at room temperature. Furthermore, storage costs and space requirements are minimized, as it is possible to preserve samples up to 360 days under most of the evaluated conditions. It is also important to note that this methodology is designed to mitigate the biological risk associated with transportation of an exotic pathogen.

## Figures and Tables

**Table 1:** Primers and probe sequences used.

Primers and Probe	Sequence (5′ to 3′)
BTV/S-9/956-978 FP1	GYRCGGGNGGDGAYRYDAARAYG
BTV/S-9/1048-1026 RP1	RWRBRAAATCGCMCTACGTCAAG
BTV/S-9/956-977 FP2	GTACAGGCGGAGATGTGAAAACG
BTV/S-9/1048-1026 RP2	GTGTAAAACCGCTATATGCCGTG
BTV/S-9/1000-1023 P	FAM-CACCTCTAA/ZEN/AGGGTCCAGGGTACC-31ABkFQ

**Table 2:** Treatments performed before RNA extraction. A total of 200 µL of hydration buffer was used for all conditions.

Condition	Card Hydration	Post-Hydration Incubation Temp	Post-Hydration Incubation Time	Agitation
*1*	TE Buffer	37 °C	30 min	No
*2*	TE Buffer	60 °C	30 min	No
*3*	LYSIS Buffer	37 °C	30 min	No
*4*	LYSIS Buffer	60 °C	30 min	No
*5*	CHELEX	60 °C	25 min	1000 rpm
*6*	TED10 + CHELEX	26 °C and then 60 °C	15 min and then 25 min	1000 rpm

Description of the conditions: 1, 2: Tris (10 mM)-EDTA (1 mM) buffer. 3, 4: RL buffer solution + RNA Carrier (TIANGEN, 1 μg/μL). 5: Chelex 100 (Bio-Rad Laboratories, Inc., Hercules, CA, USA) 5% resin solution. 6: 90% TE Buffer + 10% Dimethyl sulfoxide (Sigma-Aldrich, St. Louis, MO, USA).

**Table 3:** Trial to determine the absence of infective material on cards.

Sample ID	Sample Type	Passed Through Card
CN1	DMEM 2% FBS	No
CN2	DMEM 2% FBS	Yes
V1	Viral Stock	No
V2	Viral Stock	Yes
M1	Viral Stock + bovine blood	No
M2	Viral Stock + bovine blood	Yes

**Table 4:** Stability trial conditions, storage time, and temperature.

Condition	Storage	Days for RNA Extraction
1	−80 °C	1, 7, 30, 60, 120, and 360 days
1	−20 °C	1, 7, 30, 60, 120, and 360 days
1	4 °C	1, 7, 30, 60, 120, and 360 days
1	25 °C	1, 7, 30, 60, 120, and 360 days
2	−80 °C	1, 7, 30, 60, 120, and 360 days
2	−20 °C	1, 7, 30, 60, 120, and 360 days
2	4 °C	1, 7, 30, 60, 120, and 360 days
2	25 °C	1, 7, 30, 60, 120, and 360 days

**Table 5:** RT-qPCR results obtained after testing six different filter paper card processing conditions.

Treatment	Average Cq	Standard Deviation (σ) Cq
Hydration with TE at 37 °C for 30 min	25.02	1.08
Hydration with TE at 60 °C for 30 min	26.16	0.23
Hydration with Lysis Buffer at 37 °C for 30 min	27.41 *	1.31
Hydration with Lysis Buffer at 60 °C for 30 min	26.82 *	1.11
Hydration with CHELEX at 60 °C for 25 min	26.02	1.07
Hydration with Buffer TED10 + CHELEX at 26 °C + 60 °C for 15 min + 25 min, respectively	27.23 *	0.26

A general linear model was fitted, and orthogonal contrasts were performed between treatments using Bonferroni correction. (*) significant differences with treatment 1 (*p* < 0.05).

**Table 6:** Viral infectivity assay and RT-qPCR detection of BTV genome to evaluate the inactivation efficacy of the filter paper card system.

Sample	Passages on Vero Cells	CPE	RT-qPCR
CN1: DMEM 2% FBS	1	No	Non detectable
CN2: DMEM 2% FBS fixed on the FPC	1	No	Non detectable
V1: Viral Stock	1	Yes	Detectable
V2: Viral Stock fixed on the FPC	3	No	Non detectable
M1: Viral Stock + bovine blood	1	Yes	Detectable
M2: Viral Stock + bovine blood, fixed on the FPC	3	No	Non detectable

**Table 7:** Comparisons between treatments at different time points and their corresponding *p*-values.

Blood Spiked with BTV 1/10			Blood Spiked with BTV 1/1000		
Time	Temperature	*p*-Value	Time	Temperature	*p*-Value
1	UF −80 °C ^A^	0.1069	1	UF −80 °C ^A^	0.3373
	F −20 °C ^A^			F −20 °C ^A^	
	R 4 °C ^A^			R 4 °C ^A^	
	RT 25 °C ^A^			RT 25 °C ^A^	
7	UF −80 °C ^A^	0.2482	7	UF −80 °C ^A^	0.9492
	F −20 °C ^A^			F −20 °C ^A^	
	R 4 °C ^A^			R 4 °C ^A^	
	RT 25 °C ^A^			RT 25 °C ^A^	
30	UF −80 °C ^A^	0.3319	30	UF −80 °C ^A^	0.0418
	F −20 °C ^A^			F −20 °C ^A^	
	R 4 °C ^A^			R 4 °C ^A^	
	RT 25 °C ^A^			RT 25 °C ^B^	
60	UF −80 °C ^A^	0.4731	60	UF −80 °C ^A^	0.2827
	F −20 °C ^A^			F −20 °C ^A^	
	R 4 °C ^A^			R 4 °C ^A^	
	RT 25 °C ^A^			RT 25 °C ^A^	
120	UF −80 °C ^A^	0.2401	120	UF −80 °C ^A^	0.5771
	F −20 °C ^A^			F −20 °C ^A^	
	R 4 °C ^A^			R 4 °C ^A^	
	RT 25 °C ^A^			RT 25 °C ^A^	
360	UF −80 °C ^A^	<0.0001	360	UF −80 °C ^A^	<0.0001
	F −20 °C ^A^			F −20 °C ^A^	
	R 4 °C ^A^			R 4 °C ^A^	
	RT 25 °C ^B^			RT 25 °C ^B^	

Results are presented for two concentrations of virus diluted in blood: 1:10 and 1:1.000 dilutions, corresponding to 10^5^ and 10^3^ TCID_50_/mL, respectively. Different letters indicate significant differences (analysis performed by fitting a general linear model and comparing treatments through orthogonal contrasts).

**Table 8:** Agreement between the two viral detection techniques assessed using Cohen’s Kappa index.

	Detectable Blood	Non Detectable Blood	Total
Detectable FPC	25	2	27
Non detectable FPC	1	20	21
Total	26	22	48

## Data Availability

The original contributions presented in this study are included in the article/Supplementary Materials. Further inquiries can be directed to the corresponding author.

## Supplementary Materials

The following supporting information can be downloaded at https://www.mdpi.com/article/10.3390/vetsci13090955/s1. Supplementary Material S1: Viral stock quantification (gc/µl reaction); Supplementary Material S2: Standardization of the RNA elution from filter cards; Supplementary Material S3: Analytical sensitivity of the FPC/RT-qPCR methodology; Supplementary Material S4: Stability assay; Supplementary Material S5: Agreement between BTV detection from blood and from FPCs.

## Abbreviations

The following abbreviations are used in this manuscript:

RT	Reverse transcription
qPCR	Quantitative Polymerase chain reaction
BTV	Bluetongue virus
WOAH	World Organization for Animal Health
FPCs	Filter paper cards
TCID_50_	Tissue Culture Infectious Dose 50%
gc	Genetic copies
VL	Viral load
CPE	Cytopathic effect
FBS	Fetal bovine serum
