# Supplementary material for: A Field-Applicable Method for Bluetongue Virus Detection Using Filter Paper Cards
Source: Vet Sci. 2026 Sep 13;13(9):955. doi: 10.3390/vetsci13090955 (PMC13611841; doi:10.3390/vetsci13090955)
Supplement: Supplementary file 1 [file vetsci-13-00955-s001.zip › vetsci-4525115-supplementary.pdf]

SUPPLEMENTARY MATERIAL

S1: Viral stock quantification (gc/µl reaction)

Well	Target	Content	Sample	Cq	Starting Quantity (SQ)	
A02	BTV	Unkn	cDNA 3395	15,02	2,34E+08	
B02	BTV	Unkn	cDNA 3395	14,60	3,12E+08	
C02	BTV	Unkn	cDNA 3395	14,77	2,78E+08	
E02	BTV	Unkn	cDNA 3395	14,77	2,78E+08	**2,75E+08**
H02	BTV	NTC	NTC			
G02	BTV	NTC	NTC RT			
E11	BTV	Std	ST3	33,17	1,00E+03	
F11	BTV	Std	ST3	32,77	1,00E+03	
G11	BTV	Std	ST3	33,14	1,00E+03	
B11	BTV	Std	ST4	29,60	1,00E+04	
C11	BTV	Std	ST4	29,58	1,00E+04	
D11	BTV	Std	ST4	29,68	1,00E+04	
A11	BTV	Std	ST5	26,42	1,00E+05	
G09	BTV	Std	ST5	26,03	1,00E+05	
H09	BTV	Std	ST5	26,18	1,00E+05	
E09	BTV	Std	ST6	23,25	1,00E+06	
F09	BTV	Std	ST6	22,97	1,00E+06	
A09	BTV	Std	ST7	19,78	1,00E+07	
B09	BTV	Std	ST7	19,48	1,00E+07	
D09	BTV	Std	ST7	19,45	1,00E+07	

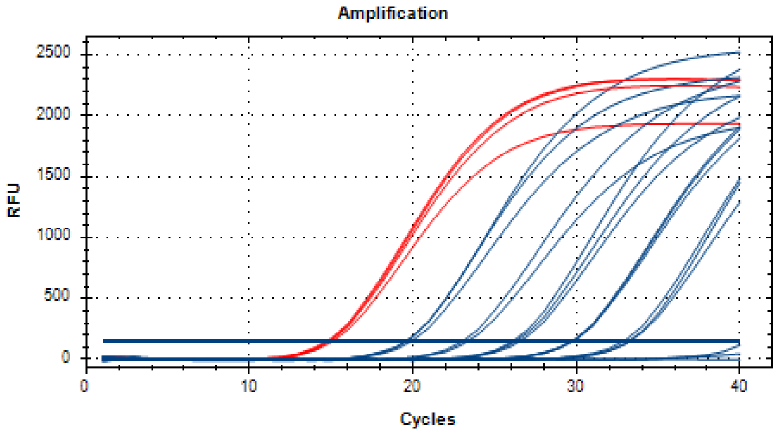

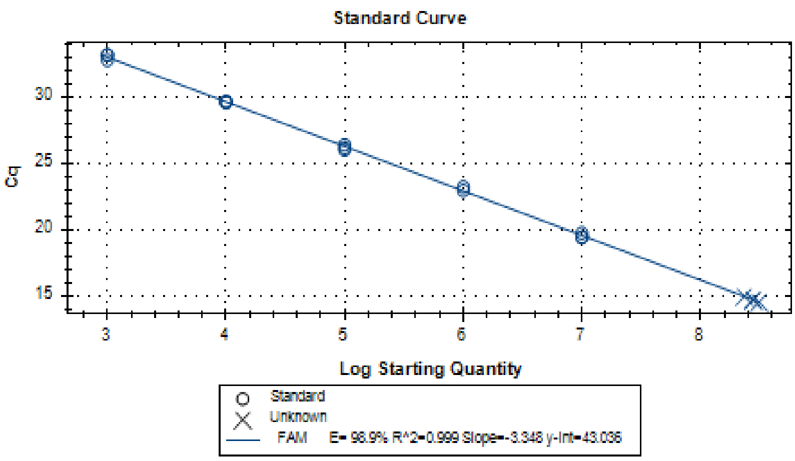

Fluor	Efficiency %	Slope	Y-Intercept	R^2
FAM	98,93	-3,348	43,036	0,999

S2: Standardization of the RNA elution from filter cards

A general linear model was fitted, and orthogonal contrasts were performed between treatments using Bonferroni correction.

**Condition**	**Card Hydration**	**Post-Hydration Incubation Temp**	**Post-Hydration Incubation Time**	**Agitation**
1	TE Buffer	37ºC	30 minutes	No
2	TE Buffer	60ºC	30 minutes	No
3	LYSIS Buffer	37ºC	30 minutes	No
4	LYSIS Buffer	60ºC	30 minutes	No
5	CHELEX[3]	60ºC	25 minutes	1000 rpm
6	TED10[4] + CHELEX	26ºC and then 60ºC	15 min and then 25 min	1000 rpm

1, 2: Tris-EDTA Buffer. 3, 4: RL buffer solution + RNA Carrier (TIANGEN, 1 µg/µl)[50]. 5: Chelex 100 5% resin solution. 6: 90% TE Buffer + 10% Dimethyl sulfoxide

Cq values for each condition

C1	C2	C3	C4	C5	C6
24.45	26.08	27.98	28.45	25	27.32
24.3	26.17	26.02	26.07	26.76	26.87
26.61	25.92	28.95	26.2	25.2	27.23
24.7	26.46	26.68	26.56	27.1	27.5

Summary statistics

Condition	Variable	n	Mean	SD	Min	Max	Median
C1	Cq	4	25.02	1.08	24.3	26.61	24.58
C2	Cq	4	26.16	0.23	25.92	26.46	26.13
C3	Cq	4	27.41	1.31	26.02	28.95	27.33
C4	Cq	4	26.82	1.11	26.07	28.45	26.38

C5	Cq	4	26.02	1.07	25	27.1	25.98
C6	Cq	4	27.23	0.26	26.87	27.5	27.28

Contrasts	p-value
C1-C2	0.0523
C1-C3	0.0113
C1-C4	0.0311
C1-C5	0.2038
C1-C6	0.0008

S3: Analytical sensitivity of the FPC/RT-qPCR methodology

A general linear model was fitted, and orthogonal contrasts between treatments were performed with Bonferroni correction.

Sample	Treatment	Dilution	Concentration (gc/µl)	Log
1	S	-1	1.33E+05	5.12
2	S	-1	1.33E+05	5.13
3	S	-1	2.80E+05	5.45
4	S	-2	4.56E+04	4.66
5	S	-2	5.46E+04	4.74
6	S	-2	3.91E+04	4.59
7	S	-2	2.47E+04	4.39
8	S	-2	4.64E+04	4.67
9	S	-3	8.76E+03	3.94
10	S	-3	5.27E+03	3.72
11	S	-3	7.08E+03	3.85
12	S	-3	5.07E+03	3.71
13	S	-3	6.30E+03	3.80
14	S	-3	8.06E+03	3.91
15	S	-3	7.54E+03	3.88
16	S	-4	7.59E+02	2.88
17	S	-4	9.59E+02	2.98
18	S	-4	1.86E+02	2.27
19	S	-4	1.80E+02	2.26
20	S	-5	2.34E+01	1.37
21	S	-5	6.47E+01	1.81
22	S	-5	5.98E+00	0.78
23	S	-5	ND	
24	S	-6	8.20E+00	0.91
25	S	-6	2.07E+01	1.32
26	S	-6	7.16E+00	0.85
27	S	-6	ND	

28	T	-1	7.69E+05	5.89
29	T	-1	4.65E+05	5.67
30	T	-1	4.51E+05	5.65
31	T	-2	6.49E+04	4.81
32	T	-2	3.55E+04	4.55
33	T	-2	7.79E+04	4.89
34	T	-2	1.98E+04	4.30
35	T	-2	7.04E+04	4.85
36	T	-2	6.23E+03	3.79
37	T	-2	7.25E+03	3.86
38	T	-3	2.61E+03	3.42
39	T	-3	1.95E+03	3.29
40	T	-3	2.88E+04	4.46
41	T	-3	2.78E+03	3.44
42	T	-3	3.60E+03	3.56
43	T	-3	3.57E+03	3.55
44	T	-3	4.22E+03	3.63
45	T	-4	1.38E+02	2.14
46	T	-4	1.12E+02	2.05
47	T	-4	1.07E+02	2.03
48	T	-4	1.37E+02	2.14
49	T	-5	6.33E+01	1.80
50	T	-5	4.26E+01	1.63
51	T	-5	3.70E+01	1.57
52	T	-5	3.79E+01	1.58
53	T	-6	ND	
54	T	-6	ND	
55	T	-6	4.49E+00	0.65
56	T	-6	7.67E+00	0.88

ND: non detectable

Summary statistics

Dilution	Treatment	Variable	n	Mean	SD	Min	Max	Median
-1	S	LOG10_Concentration	3	5.23	0.19	5.12	5.45	5.12
-1	T	LOG10_Concentration	3	5.74	0.13	5.65	5.89	5.67
-2	S	LOG10_Concentration	5	4.61	0.13	4.39	4.74	4.66
-2	T	LOG10_Concentration	7	4.44	0.46	3.79	4.89	4.55
-3	S	LOG10_Concentration	7	3.83	0.09	3.71	3.94	3.85
-3	T	LOG10_Concentration	7	3.62	0.39	3.29	4.46	3.55
-4	S	LOG10_Concentration	4	2.6	0.39	2.26	2.98	2.57
-4	T	LOG10_Concentration	4	2.09	0.06	2.03	2.14	2.09
-5	S	LOG10_Concentration	3	1.32	0.52	0.78	1.81	1.37
-5	T	LOG10_Concentration	4	1.64	0.11	1.57	1.8	1.6
-6	S	LOG10_Concentration	3	1.03	0.25	0.85	1.32	0.91
-6	T	LOG10_Concentration	2	0.77	0.16	0.65	0.88	0.77

Shapiro-Wilks (modified)

Variable	n	SD	W*	p(Unilateral D)
RE 1 LOG10_Concentration	52	0.75	0.96	0.273

Dilution	T vs S
-1	0.0185*
-2	0.3711
-3	0.1899
-4	0.0413*
-5	0.3339
-6	0.2565

S4: Stability assay

Comparisons performed between treatments at different time points. Results are presented for two concentrations of virus diluted in blood. Analysis was conducted by fitting a general linear model and comparing treatments using orthogonal contrasts

Blood spiked with BTV 1/10

SAMPLE	TIME (DAYS)	CONSERVATION	log gc/ul
1	1	F	5.55
2	1	F	5.84
3	1	F	5.22
4	1	F	5.55
5	1	F	5.58
6	1	UF	5.56
7	1	UF	5.51
8	1	UF	5.37
9	1	UF	5.20
10	1	UF	5.35
11	1	R	5.03
12	1	R	5.63
13	1	R	5.55
14	1	R	5.31
15	1	R	5.11
16	1	RT	5.54
17	1	RT	5.73
18	1	RT	5.58
19	1	RT	5.64
20	1	RT	5.56
21	7	F	5.06

22	7	F	5.64
23	7	F	5.41
24	7	F	5.45
25	7	F	5.43
26	7	UF	4.91
27	7	UF	5.04
28	7	UF	5.18
29	7	UF	
30	7	UF	
31	7	R	4.92
32	7	R	5.40
33	7	R	5.17
34	7	R	5.13
35	7	R	5.46
36	7	RT	5.23
37	7	RT	4.79
38	7	RT	5.60
39	7	RT	5.83
40	7	RT	5.73
41	30	F	5.29
42	30	F	5.06
43	30	F	5.62
44	30	F	5.51
45	30	F	5.72
46	30	UF	5.27
47	30	UF	5.67
48	30	UF	5.42
49	30	UF	5.47
50	30	UF	4.99
51	30	R	4.85
52	30	R	5.81
53	30	R	5.84
54	30	R	5.79
55	30	R	5.14
56	30	RT	5.10
57	30	RT	5.23
58	30	RT	5.16
59	30	RT	
60	30	RT	4.97
61	60	F	5.01
62	60	F	5.41
63	60	F	4.85
64	60	F	5.08
65	60	F	4.87
66	60	UF	

67	60	UF	5.74
68	60	UF	5.16
69	60	UF	
70	60	UF	5.29
71	60	R	5.08
72	60	R	5.47
73	60	R	5.22
74	60	R	5.11
75	60	R	5.63
76	60	RT	5.52
77	60	RT	5.29
78	60	RT	4.45
79	60	RT	5.45
80	60	RT	5.65
81	120	F	5.45
82	120	F	5.47
83	120	F	5.37
84	120	F	5.55
85	120	F	4.95
86	120	UF	5.07
87	120	UF	5.27
88	120	UF	4.81
89	120	UF	5.04
90	120	UF	5.09
91	120	R	
92	120	R	5.18
93	120	R	5.21
94	120	R	4.94
95	120	R	5.14
96	120	RT	4.62
97	120	RT	5.37
98	120	RT	4.93
99	120	RT	5.40
100	120	RT	5.31
101	360	F	5.68
102	360	F	5.51
103	360	F	5.38
104	360	F	5.60
105	360	F	5.86
106	360	UF	5.54
107	360	UF	5.31
108	360	UF	5.68
109	360	UF	5.85
110	360	UF	5.69
111	360	R	5.30

112	360	R	5.15
113	360	R	5.55
114	360	R	5.24
115	360	R	5.30
116	360	RT	4.65
117	360	RT	4.83
118	360	RT	4.83
119	360	RT	4.56
120	360	RT	4.41

Summary statistics

Time (days)	Conservation	n	Mean	SD	Min	Max	Median
**1**	F	5	5,55	0,22	5,22	5,84	5,55
**1**	R	5	5,33	0,26	5,03	5,63	5,31
**1**	RT	5	5,61	0,08	5,54	5,73	5,58
**1**	UF	5	5,40	0,14	5,20	5,56	5,37
**7**	F	5	5,40	0,21	5,06	5,64	5,43
**7**	R	5	5,22	0,22	4,92	5,46	5,17
**7**	RT	5	5,44	0,43	4,79	5,83	5,60
**7**	UF	3	5,04	0,14	4,91	5,18	5,04
**30**	F	5	5,44	0,27	5,06	5,72	5,51
**30**	R	5	5,49	0,46	4,85	5,84	5,79
**30**	RT	4	5,12	0,11	4,97	5,23	5,13
**30**	UF	5	5,36	0,25	4,99	5,67	5,42
**60**	F	5	5,04	0,23	4,85	5,41	5,01
**60**	R	5	5,30	0,24	5,08	5,63	5,22
**60**	RT	5	5,27	0,48	4,45	5,65	5,45
**60**	UF	3	5,40	0,30	5,16	5,74	5,29
**120**	F	5	5,36	0,24	4,95	5,55	5,45
**120**	R	4	5,12	0,12	4,94	5,21	5,16
**120**	RT	5	5,13	0,34	4,62	5,40	5,31
**120**	UF	5	5,06	0,16	4,81	5,27	5,07
**360**	F	5	5,61	0,18	5,38	5,86	5,60
**360**	R	5	5,31	0,15	5,15	5,55	5,30
**360**	RT	5	4,66	0,18	4,41	4,83	4,65
**360**	UF	5	5,61	0,20	5,31	5,85	5,68

Orthogonal contrasts

Time (days)	Orthogonal contrast	Estimate	SE	t	p value	95% CI low	95% CI high	Residual df	Bonferroni p (x3)	Significant p<0.05	Significant Bonf.
1	C1: F vs UF+R+RT	0,3100	0,2939	1,0549	0,3071	-0,3129	0,9329	16,0000	0,9214	No	No
1	C2: UF vs R+RT	-0,1400	0,2078	-0,6738	0,5101	-0,5805	0,3005	16,0000	1,0000	No	No
1	C3: R vs RT	-0,2840	0,1200	-2,3673	0,0309	-0,5383	-0,0297	16,0000	0,0926	Yes	No
7	C1: F vs UF+R+RT	0,4987	0,4526	1,1018	0,2891	-0,4720	1,4694	14,0000	0,8674	No	No
7	C2: UF vs R+RT	-0,5653	0,3744	-1,5101	0,1533	-1,3683	0,2376	14,0000	0,4598	No	No
7	C3: R vs RT	-0,2200	0,1798	-1,2233	0,2414	-0,6057	0,1657	14,0000	0,7242	No	No
30	C1: F vs UF+R+RT	0,3550	0,4821	0,7364	0,4728	-0,6725	1,3825	15,0000	1,0000	No	No
30	C2: UF vs R+RT	0,1270	0,3443	0,3688	0,7174	-0,6069	0,8609	15,0000	1,0000	No	No
30	C3: R vs RT	0,3710	0,2066	1,7958	0,0927	-0,0694	0,8114	15,0000	0,2781	No	No
60	C1: F vs UF+R+RT	-0,8387	0,5262	-1,5937	0,1333	-1,9673	0,2900	14,0000	0,4000	No	No
60	C2: UF vs R+RT	0,2193	0,4353	0,5039	0,6222	-0,7143	1,1529	14,0000	1,0000	No	No
60	C3: R vs RT	0,0300	0,2091	0,1435	0,8880	-0,4185	0,4785	14,0000	1,0000	No	No
120	C1: F vs UF+R+RT	0,7745	0,3704	2,0912	0,0539	-0,0149	1,5639	15,0000	0,1618	No	No
120	C2: UF vs R+RT	-0,1315	0,2645	-0,4971	0,6263	-0,6954	0,4324	15,0000	1,0000	No	No
120	C3: R vs RT	-0,0085	0,1587	-0,0536	0,9580	-0,3468	0,3298	15,0000	1,0000	No	No
360	C1: F vs UF+R+RT	1,2400	0,2773	4,4717	0,0004	0,6521	1,8279	16,0000	0,0012	Yes	Yes
360	C2: UF vs R+RT	1,2640	0,1961	6,4463	0,0000	0,8483	1,6797	16,0000	0,0000	Yes	Yes
360	C3: R vs RT	0,6520	0,1132	5,7593	0,0000	0,4120	0,8920	16,0000	0,0001	Yes	Yes

TIME (DAYS)	p-valor (CONSERVATION)
1	0.1069
7	0.2482
30	0.3319
60	0.4731
120	0.2401
360	<0.0001

Blood spiked with BTV 1/1000

SAMPLE	TIME (DAYS)	CONSERVATION	log gg/ul
1	1	F	3.87
2	1	F	3.81
3	1	F	3.62
4	1	F	3.99
5	1	F	3.82
6	1	UF	3.93
7	1	UF	3.81
8	1	UF	3.98
9	1	UF	3.15
10	1	UF	3.57
11	1	R	3.86
12	1	R	3.69
13	1	R	4.02
14	1	R	3.97
15	1	R	3.85
16	1	RT	4.08
17	1	RT	3.87
18	1	RT	3.90

19	1	RT	4.11
20	1	RT	3.69
21	7	F	3.53
22	7	F	3.39
23	7	F	2.94
24	7	F	2.71
25	7	F	3.57
26	7	UF	3.09
27	7	UF	2.92
28	7	UF	3.43
29	7	UF	3.03
30	7	UF	3.31
31	7	R	2.72
32	7	R	3.40
33	7	R	3.51
34	7	R	3.29
35	7	R	3.25
36	7	RT	3.15
37	7	RT	3.10
38	7	RT	3.33
39	7	RT	3.26
40	7	RT	3.41
41	30	F	3.03
42	30	F	3.48
43	30	F	3.42
44	30	F	3.58
45	30	F	3.13
46	30	UF	3.42
47	30	UF	3.47
48	30	UF	3.02
49	30	UF	3.11
50	30	UF	3.31
51	30	R	3.42
52	30	R	3.58
53	30	R	3.33
54	30	R	3.06
55	30	R	3.01
56	30	RT	2.99
57	30	RT	3.03
58	30	RT	2.96
59	30	RT	3.03
60	30	RT	2.70
61	60	F	2.87
62	60	F	3.27
63	60	F	3.40

64	60	F	3.33
65	60	F	2.46
66	60	UF	3.69
67	60	UF	3.01
68	60	UF	3.68
69	60	UF	3.34
70	60	UF	2.83
71	60	R	2.46
72	60	R	2.59
73	60	R	3.01
74	60	R	3.22
75	60	R	3.01
76	60	RT	2.99
77	60	RT	3.03
78	60	RT	2.55
79	60	RT	3.03
80	60	RT	2.92
81	120	F	3.10
82	120	F	3.46
83	120	F	3.18
84	120	F	2.93
85	120	F	3.06
86	120	UF	3.17
87	120	UF	2.90
88	120	UF	3.24
89	120	UF	3.09
90	120	UF	3.34
91	120	R	3.27
92	120	R	3.30
93	120	R	3.24
94	120	R	3.04
95	120	R	3.04
96	120	RT	3.31
97	120	RT	2.87
98	120	RT	3.47
99	120	RT	3.30
100	120	RT	3.45
101	360	F	3.47
102	360	F	3.77
103	360	F	3.53
104	360	F	3.80
105	360	F	3.45
106	360	UF	3.61
107	360	UF	3.48
108	360	UF	3.61

109	360	UF	3.68
110	360	UF	3.01
111	360	R	2.95
112	360	R	3.01
113	360	R	2.88
114	360	R	3.00
115	360	R	2.43
116	360	RT	2.43
117	360	RT	2.22
118	360	RT	2.08
119	360	RT	2.35

Summary statistics

Calibrator	Time (days)	Conservation	n	Mean	SD	Min	Max	Median
**1/100**	1	F	5	3,82	0,13	3,62	3,99	3,82
**1/100**	1	R	5	3,88	0,13	3,69	4,02	3,86
**1/100**	1	RT	5	3,93	0,17	3,69	4,11	3,90
**1/100**	1	UF	5	3,69	0,34	3,15	3,98	3,81
**1/100**	7	F	5	3,23	0,38	2,71	3,57	3,39
**1/100**	7	R	5	3,23	0,30	2,72	3,51	3,29
**1/100**	7	RT	5	3,25	0,13	3,10	3,41	3,26
**1/100**	7	UF	5	3,16	0,21	2,92	3,43	3,09
**1/100**	30	F	5	3,33	0,24	3,03	3,58	3,42
**1/100**	30	R	5	3,28	0,24	3,01	3,58	3,33
**1/100**	30	RT	5	2,94	0,14	2,70	3,03	2,99
**1/100**	30	UF	5	3,27	0,20	3,02	3,47	3,31
**1/100**	60	F	5	3,07	0,40	2,46	3,40	3,27
**1/100**	60	R	5	2,86	0,32	2,46	3,22	3,01
**1/100**	60	RT	5	2,90	0,20	2,55	3,03	2,99
**1/100**	60	UF	5	3,31	0,39	2,83	3,69	3,34
**1/100**	120	F	5	3,15	0,20	2,93	3,46	3,10
**1/100**	120	R	5	3,18	0,13	3,04	3,30	3,24
**1/100**	120	RT	5	3,28	0,24	2,87	3,47	3,31
**1/100**	120	UF	5	3,15	0,17	2,90	3,34	3,17
**1/100**	360	F	5	3,60	0,17	3,45	3,80	3,53
**1/100**	360	R	5	2,85	0,24	2,43	3,01	2,95
**1/100**	360	RT	4	2,27	0,15	2,08	2,43	2,29
**1/100**	360	UF	5	3,48	0,27	3,01	3,68	3,61

Orthogonal contrasts

Time (days)	Orthogonal contrast	Estimate	SE	t	p value	95% CI low	95% CI high	Residual df	Bonferroni p (x3)	Significant p<0.05	Significant Bonf.
1	C1: F vs UF+R+RT	-0,0300	0,3276	-0,0916	0,9282	-0,7246	0,6646	16,0000	1,0000	No	No
1	C2: UF vs R+RT	-0,4320	0,2317	-1,8647	0,0807	-0,9231	0,0591	16,0000	0,2420	No	No
1	C3: R vs RT	-0,0520	0,1338	-0,3888	0,7026	-0,3356	0,2316	16,0000	1,0000	No	No
7	C1: F vs UF+R+RT	0,0440	0,4236	0,1039	0,9186	-0,8540	0,9420	16,0000	1,0000	No	No
7	C2: UF vs R+RT	-0,1720	0,2995	-0,5742	0,5738	-0,8070	0,4630	16,0000	1,0000	No	No
7	C3: R vs RT	-0,0160	0,1729	-0,0925	0,9274	-0,3826	0,3506	16,0000	1,0000	No	No
30	C1: F vs UF+R+RT	0,4960	0,3206	1,5470	0,1414	-0,1837	1,1757	16,0000	0,4242	No	No
30	C2: UF vs R+RT	0,3100	0,2267	1,3674	0,1904	-0,1706	0,7906	16,0000	0,5712	No	No
30	C3: R vs RT	0,3380	0,1309	2,5823	0,0200	0,0605	0,6155	16,0000	0,0601	Yes	No
60	C1: F vs UF+R+RT	0,1260	0,5201	0,2423	0,8117	-0,9766	1,2286	16,0000	1,0000	No	No
60	C2: UF vs R+RT	0,8580	0,3678	2,3330	0,0330	0,0784	1,6376	16,0000	0,0991	Yes	No
60	C3: R vs RT	-0,0460	0,2123	-0,2166	0,8312	-0,4961	0,4041	16,0000	1,0000	No	No
120	C1: F vs UF+R+RT	-0,1680	0,2914	-0,5764	0,5723	-0,7858	0,4498	16,0000	1,0000	No	No
120	C2: UF vs R+RT	-0,1620	0,2061	-0,7861	0,4433	-0,5989	0,2749	16,0000	1,0000	No	No
120	C3: R vs RT	-0,1020	0,1190	-0,8573	0,4040	-0,3542	0,1502	16,0000	1,0000	No	No
360	C1: F vs UF+R+RT	2,2100	0,3414	6,4728	0,0000	1,4823	2,9377	15,0000	0,0000	Yes	Yes
360	C2: UF vs R+RT	1,8320	0,2439	7,5119	0,0000	1,3122	2,3518	15,0000	0,0000	Yes	Yes
360	C3: R vs RT	0,5840	0,1463	3,9911	0,0012	0,2721	0,8959	15,0000	0,0035	Yes	Yes

TIME (DAYS)	p-valor (CONSERVATION)
1	0.3373
7	0.9492
30	0.0418
60	0.2827
120	0.5771
360	<0.0001

Since independent filter paper cards were processed at each sampling time, observations were considered independent and a repeated-measures design was not applicable. The effects of storage time and temperature on viral genome stability were therefore evaluated using a two-way factorial general linear model, including time, temperature, and the time × temperature interaction. Analyses were performed separately for each viral concentration, considering time as categoric factor.

$\log_{10}$ gc/µL ~ Time + Temperature + Time × Temperature

BTV dilution	time	temperature	Time x temperature
1/10	p= 0.0119	p= 0.0474	p= 0.000016
1/100	p<0.0001	p= 0.00045	p>0.0000001

S5. Agreement between BTV detection from blood and from FPCs

Sample ID	Sample type	Specie	Cq value*	qPCR result	Ab ELISA result
BOV LH1	blood	bovine	Undetermined	ND	Positive
BOV LH1	FPC	bovine	Undetermined	ND	nd
BOV LH2	blood	bovine	Undetermined	ND	Positive
BOV LH2	FPC	bovine	Undetermined	ND	nd

BOV LH3	blood	bovine	Undetermined	ND	Negative
BOV LH3	FPC	bovine	Undetermined	ND	nd
BOV LH4	blood	bovine	33,60	DETECTABLE	Positive
BOV LH4	FPC	bovine	34,62	DETECTABLE	nd
BOV LH5	blood	bovine	34,38	DETECTABLE	Positive
BOV LH5	FPC	bovine	36,51	DETECTABLE	nd
BOV LH6	blood	bovine	Undetermined	ND	Positive
BOV LH6	FPC	bovine	Undetermined	ND	nd
BOV LH11	blood	bovine	34,19	DETECTABLE	Positive
BOV LH11	FPC	bovine	37,24	DETECTABLE	nd
BOV LH12	blood	bovine	31,42	DETECTABLE	Positive
BOV LH12	FPC	bovine	29,62	DETECTABLE	nd
BOV LH13	blood	bovine	33,96	DETECTABLE	Positive
BOV LH13	FPC	bovine	35,37	DETECTABLE	nd
BOV LH16	blood	bovine	Undetermined	ND	Positive
BOV LH16	FPC	bovine	Undetermined	ND	nd
BOV LH19	blood	bovine	37,66	DETECTABLE	Positive
BOV LH19	FPC	bovine	38	DETECTABLE	nd
BOV LH22	blood	bovine	Undetermined	ND	Positive
BOV LH22	FPC	bovine	Undetermined	ND	nd
BOV LH23	blood	bovine	Undetermined	ND	Positive
BOV LH23	FPC	bovine	Undetermined	ND	nd
BOV LH27	blood	bovine	38,65	DETECTABLE	Positive
BOV LH27	FPC	bovine	39,6	DETECTABLE	nd
BOV LH28	blood	bovine	Undetermined	ND	Negative
BOV LH28	FPC	bovine	Undetermined	ND	nd
BOV LH29	blood	bovine	39,91	DETECTABLE	Negative
BOV LH29	FPC	bovine	Undetermined	ND	nd
BOV LH32	blood	bovine	Undetermined	ND	Positive
BOV LH32	FPC	bovine	Undetermined	ND	nd
BOV LH33	blood	bovine	33,52	DETECTABLE	Positive
BOV LH33	FPC	bovine	30,29	DETECTABLE	nd
BOV LH34	blood	bovine	Undetermined	ND	Positive
BOV LH34	FPC	bovine	Undetermined	ND	nd
BOV LH37	blood	bovine	37,19	DETECTABLE	Positive
BOV LH37	FPC	bovine	38,42	DETECTABLE	nd
BOV LH39	blood	bovine	Undetermined	ND	Positive
BOV LH39	FPC	bovine	Undetermined	ND	nd
BOV LH41	blood	bovine	Undetermined	ND	Positive
BOV LH41	FPC	bovine	Undetermined	ND	nd
BOV LH42	blood	bovine	Undetermined	ND	Negative
BOV LH42	FPC	bovine	Undetermined	ND	nd
BOV LH43	blood	bovine	Undetermined	ND	Positive
BOV LH43	FPC	bovine	39,49	DETECTABLE	nd
BOV LH44	blood	bovine	Undetermined	ND	Positive

BOV LH44	FPC	bovine	38,45	DETECTABLE	nd
BOV LH9	blood	bovine	37,18	DETECTABLE	Positive
BOV LH9	FPC	bovine	38,43	DETECTABLE	nd
OV LH1	blood	ovine	Undetermined	ND	Negative
OV LH1	FPC	ovine	Undetermined	ND	nd
OV LH2	blood	ovine	Undetermined	ND	Negative
OV LH2	FPC	ovine	Undetermined	ND	nd
OV LH3	blood	ovine	Undetermined	ND	Positive
OV LH3	FPC	ovine	Undetermined	ND	nd
OV LH4	blood	ovine	Undetermined	ND	Positive
OV LH4	FPC	ovine	Undetermined	ND	nd
OV VC10	blood	ovine	33,09	DETECTABLE	Positive
OV VC10	FPC	ovine	37,9	DETECTABLE	nd
OV VC11	blood	ovine	39,75	DETECTABLE	Negative
OV VC11	FPC	ovine	38,06	DETECTABLE	nd
OV VC12	blood	ovine	38,13	DETECTABLE	Positive
OV VC12	FPC	ovine	37,9	DETECTABLE	nd
OV VC13	blood	ovine	33	DETECTABLE	Positive
OV VC13	FPC	ovine	31,39	DETECTABLE	nd
OV VC15	blood	ovine	31,62	DETECTABLE	Positive
OV VC15	FPC	ovine	33,03	DETECTABLE	nd
OV VC17	blood	ovine	Undetermined	ND	Negative
OV VC17	FPC	ovine	Undetermined	ND	nd
OV VC18	blood	ovine	37,41	DETECTABLE	Positive
OV VC18	FPC	ovine	36,73	DETECTABLE	nd
OV VC19	blood	ovine	34,7	DETECTABLE	Positive
OV VC19	FPC	ovine	35,09	DETECTABLE	nd
OV VC21	blood	ovine	39,26	DETECTABLE	Negative
OV VC21	FPC	ovine	38,9	DETECTABLE	nd
OV VC22	blood	ovine	35,97	DETECTABLE	Positive
OV VC22	FPC	ovine	35,05	DETECTABLE	nd
OV VC23	blood	ovine	33,47	DETECTABLE	Positive
OV VC23	FPC	ovine	37,24	DETECTABLE	nd
OV VC28	blood	ovine	36,84	DETECTABLE	Negative
OV VC28	FPC	ovine	39,55	DETECTABLE	nd
OV VC30	blood	ovine	36,82	DETECTABLE	Positive
OV VC30	FPC	ovine	35,2	DETECTABLE	nd
OV VC31	blood	ovine	36,55	DETECTABLE	Positive
OV VC31	FPC	ovine	36,17	DETECTABLE	nd
OV VC34	blood	ovine	Undetermined	ND	Negative
OV VC34	FPC	ovine	Undetermined	ND	nd
OV VC35	blood	ovine	Undetermined	ND	Negative
OV VC35	FPC	ovine	Undetermined	ND	nd
OV VC37	blood	ovine	33,8	DETECTABLE	Negative
OV VC37	FPC	ovine	36,1	DETECTABLE	nd

OV VC38	blood	ovine	32,17	DETECTABLE	Positive
OV VC38	FPC	ovine	36,77	DETECTABLE	nd

(*) Cq values reported represent the mean Cq of the three RT-qPCR replicates

The agreement between the two viral detection techniques was assessed using Cohen's Kappa index

	DECTABLE BLOOD	ND BLOOD	TOTAL
DETECTABLE FPC	25	2	**27**
ND FPC	1	20	**21**
TOTAL	**26**	**22**	**48**

Kappa de Cohen index= 0.874 (IC 95%: 0.735 – 1.000)
